# Prevalence of Temporomandibular Disorders in Adult Women with Endometriosis

**DOI:** 10.3390/jcm13247615

**Published:** 2024-12-13

**Authors:** Tomasz Marciniak, Natalia Walewska, Agata Skoworodko, Patrycja Bobowik, Weronika Kruk-Majtyka

**Affiliations:** 1 Faculty of Rehabilitation, Józef Piłsudski Academy of Physical Education in Warsaw, 00-968 Warsaw, Poland; 2 Fizjoklinika Warszawskie Centrum Rehabilitacji i Osteopatii Medycznej, 02-002 Warsaw, Poland

**Keywords:** endometriosis, temporomandibular disorders, pelvic pain, orofacial pain, central sensitization, 3Q/TMD, TMD pain screener, DC/TMD

## Abstract

**Background/Objectives:** The prevalence of endometriosis varies between 10% and 18%, while temporomandibular disorders (TMDs) concern between 29 and 34% of the general population. Both conditions share similar etiological factors and symptoms such as widespread, chronic pain. Therefore, both are qualified as Chronic Overlapping Pain Conditions. Even though TMDs and endometriosis appear to be comorbidities, up until now, no research has examined how the incidence rates compare between them. Thus, this study aimed to analyze the prevalence of TMD symptoms in women with endometriosis in the Polish population. **Methods:** 163 adult women with endometriosis, aged 32.41 ± 6.76 years, completed an anonymous online survey regarding their medical history and TMD symptoms. The participants were screened for TMD symptoms using two questionnaires—3Q/TMD and TMD Pain Screener (part of the DC/TMD protocol). The history mainly consisted of a chronology of symptoms’ appearance, medical consultations, and final confirmation of the diagnosis, to establish delay time. **Results:** The analysis revealed that 77.3% of women with endometriosis showed TMD symptoms, and 49.08% of the whole studied population showed important pain levels. Then, the sample was divided into two groups according to the 3Q/TMD questionnaire—a TMD and an nTMD group. The results showed significantly higher pain levels in the TMD group (r = 0.721) compared to non-symptomatic subjects. The mean patients’ delay time (T1) was 2.81 ± 4.40 years, and the mean doctors’ delay (T2) was 5.32 ± 5.65 years. **Conclusions:** The results provide a new insight into the relationship between endometriosis and TMD. The prevalence of the latter condition was found to be high, creating a strong recommendation for the use of TMD screening tools in this particular population.

## 1. Introduction

“Temporomandibular disorders” (TMD) are one of the most common causes of orofacial pain [1]. This umbrella term envelops symptoms such as pain of the masticatory muscles, intra-articular disorders manifested as temporomandibular joint (TMJ) sounds, and TMJ pain, known as arthralgia [2]. The prevalence of TMD symptoms in the general population is between 29 and 34% [3,4]. In Europe, it is estimated to be 29% [4]. The etiology of TMD is multifactorial, and includes a wide range of physical and psychoemotional factors, such as emotional stress, depression, anxiety, hormonal changes, occlusion, etc. [5,6]. Temporomandibular joint problems, including osteoarthritis, are conditions that often occur in women over the age of 40, and are linked to the loss of estrogen [7].

Endometriosis is an estrogen-dependent inflammatory disease in which endometrial tissue appears outside its natural site [8], with the main symptom being pelvic pain, but also in other areas. Moreover, endometriosis is also defined as the presence of endometrial glands and stroma outside the uterine cavity [9]. It is worth mentioning that 17–44% of women diagnosed with endometriosis have an endometrioma [10,11]. Endometriosis affects approximately 5–10% of the female population, with a higher incidence in women with infertility (20–30%) [12]. Depending on the source, the mean prevalence of endometriosis in the general population varies between 10% and 18%, while in Europe it averages 17% [13]. It is stated that as many as 176–190 million women could be suffering from endometriosis-related symptoms around the globe [14,15,16]. In Poland, reliable data do not exist. It is approximated that one million women suffer from endometriosis, but the available scientific data are out of date [17].

The etiology of temporomandibular disorders and endometriosis is multifactorial, and both of the conditions share the same factors, such as emotional stress, depression, anxiety, etc. These psychoemotional features are strongly linked to the subjective perception of pain, and potentially lead to chronicity [18,19,20,21].

Moreover, it has been shown that endometriosis and TMD are examples of Chronic Overlapping Pain Conditions (COPCs), and coincide with other conditions such as chronic lower back pain, chronic tension-type headache, migraines, fibromyalgia, and chronic fatigue syndrome [7]. The literature shows that both TMD and endometriosis can manifest similar somatic symptoms outside of the original area of pathology [7]. For example, women with endometriosis commonly suffer from orofacial pain and headaches, while subjects with TMD can also present signs of widespread pain [7,22,23,24,25]. The literature concludes that having one condition from the COPC spectrum makes a person more susceptible to developing another [26].

An argument supporting this premise could lie in the central sensitization (CS) mechanism, since it plays an important role in the onset, maintenance, and chronicity of pain. Commonly, endometriosis and temporomandibular disorders present this phenomenon [27,28,29].

While previous studies have shown comorbidity between endometriosis and TMD, specific prevalence data regarding this relationship have yet to be reported. Thus, this study aimed to investigate the prevalence of TMD symptoms in Polish women with endometriosis.

## 2. Materials and Methods

### 2.1. Material

**Figure 1:**
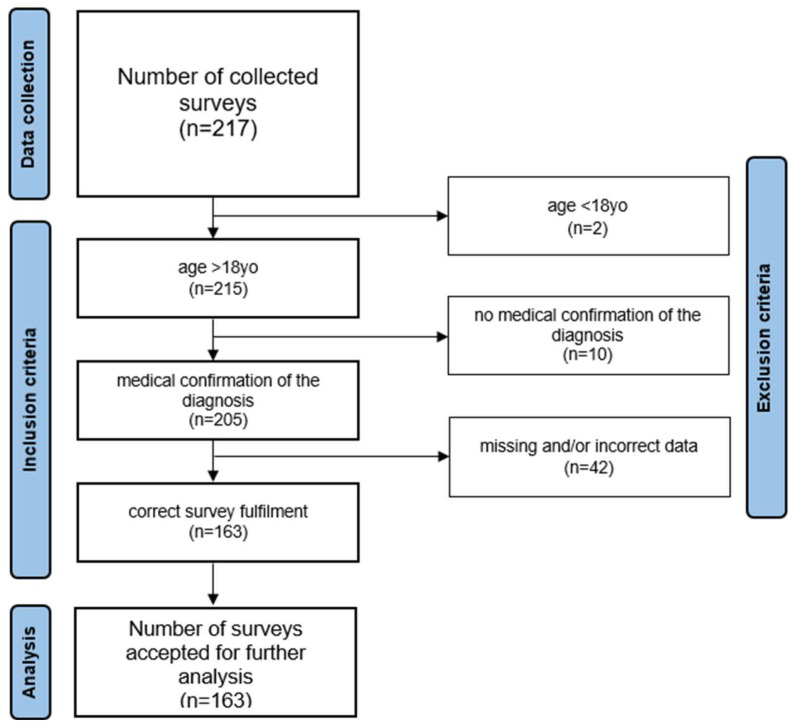
Data collection process.

A survey was completed by 217 Polish women. The inclusion criteria consisted of an age above 18 years old and a confirmation of a diagnosis of endometriosis made by a medical doctor. The study excluded those participants who did not fulfill the above-mentioned criteria, as well as those who failed to fill out the questionnaire correctly. As many as 54 women did not meet the inclusion criteria or did not fill out the survey correctly. Ultimately, 163 adult women aged 32.41 ± 6.76 years with a confirmed diagnosis of endometriosis took part in the study. The detailed data collection process is depicted in Figure 1.

The local ethical commission approved the study (signature SKE 01-14/2024) after revisions were made to ensure that the standards of the Helsinki Declaration were met.

### 2.2. Methods

The study was conducted using an anonymous online survey promoted on the internet (social media, as well as different profiles and groups related to endometriosis). The subjects volunteered to participate in the study by completing an online survey. The study was conducted between March and April 2024. The survey was divided into two parts. First, the participants were asked to fill in the demographic data and necessary medical information to see if they met the inclusion criteria. If so, they were asked to give more details about their history of endometriosis, chronology of their first symptoms, medical consultations, treatment strategies including surgeries, etc. Among others, the participants were asked the following questions:

1. When did your first symptoms of endometriosis appear? Provide the date (month and year).
2. How long after the first symptoms of endometriosis appeared did you go to the doctor? Provide the date (month and year).
3. How long after your first medical consultation did you receive a confirmed diagnosis of endometriosis? Provide the date (month and year).

The second part consisted of two TMD screening questionnaires, i.e., 3Q/TMD and TMD Pain Screener, with the latter being part of the DC/TMD protocol [2,30,31]. The questionnaires were followed by a history of trauma in the head and neck area, orthodontic and/or orthognathic treatment. The complete survey is available in Appendix A.

### 2.3. Data Analysis

All other data were analyzed using STATISTICA 14.0 software (StatSoft). The normality of the distributions of the study variables in the groups was tested using the Shapiro–Wilk test. The homogeneity of variance was assessed using Levene’s test. The Mann–Whitney U test and Kruskal–Wallis’s test were used to determine differences between groups. A significance level of α = 0.05 was adopted.

## 3. Results

### 3.1. Endometriosis

The three questions mentioned above allowed the authors to calculate the time that elapsed from the appearance of the first symptoms to the first medical consultation (T_1_), as well as from that consultation to the definite confirmation of the diagnosis of endometriosis (T_2_). The analysis revealed that the mean T_1_ was 2.81 ± 4.40 years, and the mean T_2_ was 5.32 ± 5.65 years.

Another aspect of the analysis was related to surgeries for endometriosis, along with the medical history of each patient. The results showed that 84 women (51.53%) had undergone at least one surgery as a treatment modality, versus 79 women (48.47%) who had never had this kind of procedure performed. The mean number of surgeries in the operated group was 1.33 ± 0.94.

### 3.2. Prevalence of Temporomandibular Disorders in the Population of Women with Endometriosis

The results of the prevalence of TMD symptoms in women with endometriosis were taken from the 3Q/TMD and TMD Pain Screener questionnaires. The cut-off points used in this paper followed those put forward by others [2,31,32,33].

#### 3.2.1. 3Q/TMD Questionnaire

The analysis showed that 77.3% of the participants marked at least one positive answer indicating a possible presence of temporomandibular disorders. The highest number of participants (55 subjects, 33.74%) marked all three questions as yes. On the other hand, only 22.7% of subjects responded no to all the listed questions. Detailed results are presented in Table 1.

#### 3.2.2. TMD Pain Screener Questionnaire

The analysis showed that 49.08% of all participants presented important pain levels using the TMD Pain Screener. Positivity for signs of pain is indicated by obtaining three or more points out of seven. A little more than half of the subjects, i.e., 50.92%, obtained between zero and two points, making the total screening result negative. Detailed data are presented in Table 2.

#### 3.2.3. Orthodontic and Orthognathic Treatment and Trauma

Most of the studied women with endometriosis (57.06%) had not undergone any orthodontic treatment. Of those who were receiving it at the moment of the study, or had had it in the past (70 subjects), 80% (56 subjects) reported TMD symptoms. Most of them, i.e., 41.07% (23 subjects), suffered from TMD symptoms after the course of wearing braces, and in 35.48% (22 subjects), symptoms occurred before starting orthodontics. Only 20% (14 subjects) did not report having any TMD symptoms, despite currently undergoing or having undergone orthodontic treatment. Detailed results are presented in Table 3.

Proceeding to the relationship with orthognathic treatment, only three subjects (1.84% of the studied population) stated that they had undergone surgery, but they did not complain of any pre-op or post-op TMD symptoms.

Trauma in the face and head area was reported by five subjects, which constituted 3.07% of all the participants included in the study.

### 3.3. The Relationship Between 3Q/TMD and TMD Pain Screener Results

Since a positive correlation between 3Q/TMD and Pain Screener levels was revealed, and marked as very high (r = 0.721), the authors followed the statistical analysis by examining significant differences in the TMD Pain Screener levels between the TMD and the nTMD groups (*p* < 0.001).

Furthermore, the TMD group was divided into four subgroups, according to the assessment of their 3Q/TMD scores as well as their Pain Screener (PS) levels; these are presented below. In the first subgroup (TMD-0), consisting of 37 women, the mean PS value was 0.675 ± 1.11, with Me = 0 and IC95% = 0.9–1.438. In the second subgroup (TMD-1), consisting of 37 women, the PS value was 2.189 ± 1.47, with Me = 2 and IC95% = 0.195–1.908. In the third subgroup (TMD-2), consisting of 34 women, the PS value was 2.971 ± 1.62, with Me = 2 and IC95% = 1.309–2.137. Lastly, in the fourth subgroup (TMD-3), consisting of 55 women, the PS value was 4.672 ± 1.7, with Me = 5 and IC95% = 1.431–2.094.

Finally, there were significant differences in Pain Screener levels between all the subgroups, except between TMD-1 and TMD-2 (*p* = 0.657). Detailed data are presented in Table 4.

## 4. Discussion

### 4.1. Temporal Aspect of the Diagnostic Process of Endometriosis in the General Population

Even though the prevalence of endometriosis in the general population is high, the diagnostic process is difficult and very complex, and leads to a common phenomenon called diagnostic delay, which has been described extensively by experts in the field of endometriosis [34,35]. The total delay consists of three main components: the patient’s delay, the general practitioner’s (GP) delay, and the specialist’s delay (i.e., gynecologist).

Several studies have shown that the mean time between the occurrence of the first symptoms and the first GP visit is one year (range 0–4 years) [36,37]. This time would be considered as a patient’s delay, and it obviously varies between populations from different countries. In the present research, in which Polish women were studied, the mean patient delay time (T_1_) was found to be almost three years (2.81 ± 4.40 years), which is a lot longer in comparison with studies from other countries [36,37].

Another temporal aspect of the analysis, the time between the first GP consultation and the confirmation of the diagnosis by a gynecologist (i.e., GP and specialist delay), also varies between countries. For example, in Ireland and Belgium, the total mean delay time is 4–5 years [38]; in Canada, 5.4 years [34]; in Norway, 6.7 years [39]; in the Netherlands 7.4 years [36]; in Italy 7–10 years [38]; and in Germany and Austria, 10 years [38]. In Poland, the latest reliable data come from 2012, and outline 12 years as the mean time, setting the number in the upper range of the spectrum [40]. Thus, it can be approximated that the mean time for diagnosis varies between 4 and 11 years, depending on the country [41,42].

The present research shows that the mean doctor’s delay time (GP and specialist delay time taken together) for the confirmation of the diagnosis (T_2_) was 5.32 ± 5.65 years, putting it at the lower end of the range listed above.

Despite the differences in total diagnostic time between populations, the proportional difference between the patient’s delay and the doctor’s delay seems comparable with the results provided by Staal et al. [36], in which the GP’s delay together with the specialist’s delay time was much longer than the patient’s delay. This consitutes another argument emphasizing the complexity of the diagnostic process of endometriosis.

The main struggle encountered by the authors of this study while analyzing and comparing the results was the lack of current data in the existing literature on prevalence, as well as on the time that elapses from the occurrence of the first symptoms, through GP consultation, until the final diagnosis confirmation, due to the diagnostic delay phenomenon [21,26]. The second difficulty lay within the differences in data collection methods, and the presence or absence of distinction between the individual types of delay used throughout the articles.

### 4.2. Prevalence of Temporomandibular Disorders in Women with Endometriosis

Since, to the best knowledge of the authors, this is the first study concerning the subject of the prevalence of TMD symptoms in women with endometriosis, there are therefore no available data for a direct comparison of the incidence rate.

In the current study, the authors found that more than 77% of women diagnosed with endometriosis fulfilled the screening criteria for TMD using the 3Q/TMD questionnaire, setting the prevalence rate very high.

The latest study conducted by Wójcik et al. [7] confirmed the relation between endometriosis and temporomandibular joint pain. They concluded that there was a significant correlation between the presence of pain on the right and left sides of the pelvis, and pain on the right and left sides of the temporomandibular joint (*p*-value = 0.0397, V = 0.2350). Moreover, there was a correlation between the occurrence of pelvic pain and the treatment modality for endometriosis (*p*-value = 0.0104, V = 0.3709). Interestingly, there was no relationship between the occurrence of temporomandibular joint pain and the treatment of endometriosis (*p*-value = 0.5214, V = 0.3274). The authors used a self-made survey in which women with endometriosis were asked about different aspects related to their symptoms, among others regarding orofacial pain, clenching, headaches, endometriosis treatment modalities, etc. Even though they used independent measurement tools, they reached similar outcomes confirming the comorbidity. Despite all the mentioned findings, the authors did not estimate the prevalence of TMD symptoms in the studied sample.

Since the main complaint of patients with both conditions is pain, the authors of the current study performed a statistical analysis, which revealed a positive correlation between 3Q/TMD and Pain Screener levels in the studied population. Moreover, there were significant differences in pain levels between the TMD and nTMD groups. The results led to a conclusion that women with endometriosis who also present signs of temporomandibular disorders experience higher levels of pain compared to subjects without TMD.

### 4.3. Links Between Temporomandibular Disorders and Endometriosis

From a scientific and a clinical point of view, endometriosis and temporomandibular disorders are linked by several mechanisms.

The first potential mechanism underlying the phenomenon of pain co-occurrence in both conditions may be the fact that endometriosis and TMD are multifactorial and share mutual etiology, such as emotional stress, depression, anxiety, catastrophizing, and kinesiophobia, which influence and multiply pain experience [18,19,21,43].

Bearing in mind the second factor, that endometriosis and TMD form part of Chronic Overlapping Pain Conditions (COPCs), it seems crucial to remember that widespread pain is very common in both populations. It has been shown that people with one COPC have an increased likelihood of having another COPC, and the perception of pain is likely to be further amplified [20,26,27]. Pain amplification and psychosocial vulnerability are considered the two major interactive domains contributing to the development and maintenance of COPCs [26]. In the current study, neither of the pain-contributing factors mentioned earlier had been assessed. Moreover, there is growing evidence showing comorbidity between endometriosis and other musculoskeletal disorders [7]. It should be noted that endometriosis does not have the pathognomonic symptoms characteristic of isolated pelvic disease [42]. The manifestation of pain in the orofacial, head, and neck regions especially concerns TMD, including headaches, migraines, and bruxism [7,23].

The last factor explaining the possible link states that both of the conditions share a tendency for chronicity, and are prone to central sensitization (CS) development. Recent studies have shown that the hypersensitivity of the nervous system when processing nociceptive input plays a major role in the onset and maintenance of pain. In many cases, central sensitization is the basis for chronic pain [29]. CS has a high prevalence in patients with endometriosis, especially in those with moderate to severe chronic pelvic pain, presenting three central sensitivity syndromes (i.e., migraine or tension-type headache, irritable bowel syndrome, and anxiety or panic attacks) [28].

All the described factors and mechanisms explain the link between temporomandibular disorders and endometriosis, justifying the findings of the current study.

The theoretical considerations conclude that a clear practical and clinical indication should be addressed to all medical professionals inside the multidisciplinary team who treat endometriosis patients, and highlight the importance of performing a screening examination for orofacial pain and TMD symptoms. Even though the ESHRE guidelines on diagnosing endometriosis do not mention orofacial pain or headaches (but only shoulder pain) as potential indicators for a diagnosis of endometriosis, the presented links seem to play an important role [44].

### 4.4. Study Limitations

The authors observed some study limitations. One limitation was that the data were collected via an online survey, which could influence the quality of the results, even though it was the best manner in which to screen and cross-sectionally examine the population. Another limitation was the sample size.

## 5. Conclusions

The prevalence of TMD symptoms in women with endometriosis is high, reaching up to 77.3% of the studied population. Also, women with both the conditions of endometriosis and TMD showed significantly higher pain levels. Considering these results, there is a strong recommendation for the use of TMD screening tools in women with symptoms and/or a diagnosis of endometriosis.

Future research should focus on bigger samples from different countries, as well as onsite examination using more detailed tools, such as the DC/TMD protocol, which also includes the psychoemotional status of the subjects.

## Figures and Tables

**Table 1:** Temporomandibular Disorder prevalence in women with endometriosis, according to the 3Q/TMD questionnaire.

3Q/TMD Positive Answers n = 163	n	%	Interpretation
0	37	22.7	nTMD
1	37	22.7	TMD
2	34	20.86	
3	55	33.74	

**Table 2:** TMD Pain Screener questionnaire results in women with endometriosis.

TMD Pain Screener Points n = 163	n	%	Interpretation
0	26	15.95	TMD pain negative
1	29	17.79	
2	28	17.18	
3	16	9.82	TMD pain positive
4	20	12.27	
5	20	12.27	
6	17	10.43	
7	7	4.29	

**Table 3:** Orthodontic treatment history and appearance of TMD symptoms in the whole studied population of women with endometriosis.

Orthodontic Treatment Status n = 163	TMD Symptom Appearance									
			Before		During		After		nTMD	
	n	%	n	%	n	%	n	%	n	%
no orthodontic treatment	93	57.06	-	-	-	-	-	-	-	-
during orthodontic treatment	8	4.91	5	62.5	1	12.5	-	-	2	25
orthodontic treatment finished	62	38.03	22	35.48	5	8.06	23	37.1	12	19.35
**Total**			**56 TMD subjects** **(80%)**						**14 nTMD subjects (20%)**	

**Table 4:** TMD Pain Screener level differences between 3Q/TMD subgroups.

	TMD-1	TMD-2	TMD-3
**TMD-0**	0.003	<0.001	<0.001
**TMD-1**	-	0.657	<0.001
**TMD-2**	0.657	-	0.006

## Data Availability

The data are available on request from the corresponding author.

## Appendix A. The Online Survey

**Initial data (inclusion criteria)**
  1. Are you of adult age (18+ years old)?
    - Yes
    - No
  2. How old are you?
  3. Do you have a confirmed diagnosis of endometriosis by a medical doctor?
    - Yes
    - No
**Endometriosis—history**
  4. When did your first symptoms of endometriosis appear? Provide the date (month and year).
  5. How long after the first symptoms of endometriosis appeared did you go to the doctor? Provide the date (month and year).
  6. How long after your first medical consultation did you receive a confirmed diagnosis of endometriosis? Provide the date (month and year).
  7. Have you undergone surgical treatment for endometriosis?
    - Yes
    - No
  8. When did you have the surgery?
**Temporomandibular disorders—history**
  **3Q/TMD Questionnaire**
  1. Do you have pain in your temple, face, jaw, or jaw joint once a week or more?
    - Yes
    - No
  2. Do you have pain once a week or more, when you open your mouth or chew?
    - Yes
    - No
  3. Does your jaw lock or become stuck once a week or more?
    - Yes
    - No
**TMD Pain Screener**
  1. In the last 30 days, how long did any pain last in your jaw or temple area on either side?
    - No pain
    - Pain comes and goes
    - Pain is always present
  2. In the last 30 days, have you had pain or stiffness in your jaw on awakening?
    - No
    - Yes
  3. In the last 30 days, did the following activities change any pain (that is, make it better or make it worse) in your jaw or temple area on either side?
    A. Chewing hard or tough food
    a. No
    b. Yes
    B. Opening your mouth or moving your jaw forward or to the side
    a. No
    b. Yes
    C. Jaw habits such as holding teeth together, clenching, grinding, or chewing gum
    a. No
    b. Yes
    D. Other jaw activities such as talking, kissing, or yawning
    a. No
    b. Yes
**Orthodontic treatment, orthognathic treatment, and craniofacial injuries—history**
  1. Have you ever had or are you currently undergoing orthodontic treatment? (braces or overlay treatment)
    - I have had orthodontic treatment
    - I am undergoing orthodontic treatment
    - I have not had orthodontic treatment
  2. When did you start (or start and finish) orthodontic treatment?
  3. Did the above-mentioned symptoms of temporomandibular disorders (TMD) appear before, during, or after orthodontic treatment?
    - Before treatment
    - During treatment
    - After treatment
    - Not applicable
  4. Have you had orthognathic treatment (as part of maxillofacial surgery) in the past?
    - Yes
    - No
  5. When did you have an orthognathic surgery? Provide the date (month and year).
  6. Did the above-mentioned symptoms of temporomandibular disorder (TMD) appear before or after orthognathic treatment?
    - Before treatment
    - After treatment
  7. Have you ever experienced an injury (e.g., fracture) in the face/head area?
    - Yes
    - No
  8. When did the injury occur? Provide the date (month and year).
  9. Did the above-mentioned symptoms of temporomandibular disorder (TMD) appear before or after the injury?
    - Before the injury
    - After the injury
